# Fine Mapping of *BoVl* Conferring the Variegated Leaf in Ornamental Kale (*Brassica oleracea* var. *acephala*)

**DOI:** 10.3390/ijms232314853

**Published:** 2022-11-27

**Authors:** Jie Ren, Jiaqi Zou, Xiao Zou, Gengxing Song, Zhichao Gong, Zhiyong Liu, Ruiqin Ji, Hui Feng

**Affiliations:** Liaoning Key Laboratory of Genetics and Breeding for Cruciferous Vegetable Crops, College of Horticulture, Shenyang Agricultural University, Shenyang 110065, China

**Keywords:** ornamental kale, variegated leaf, BSR-seq, chloroplast, transcriptome

## Abstract

Ornamental kale, as a burgeoning landscaping plant, is gaining popularity for its rich color patterns in leaf and cold tolerance. Leaf variegation endows ornamental kale with unique ornamental characters, and the mutants are ideal materials for exploring the formation mechanisms of variegated phenotype. Herein, we identified a novel variegated leaf kale mutant ‘JC007-2B’ with green margins and white centers. Morphological observations and physiological determinations of the green leaf stage (S1), albino stage (S2) and variegated leaf stage (S3) demonstrated that the chloroplast structure and photosynthetic pigment content in the white sectors (S3_C) of variegated leaves were abnormal. Genetic analysis revealed that a single dominant nuclear gene (*BoVl*) controlled the variegated leaf trait of ‘JC007-2B’, and three candidate genes for *BoVl* were fine-mapped to a 6.74 Kb interval on chromosome C03. Multiple sequence alignment among the green-leaf mapping parent ‘BS’, recombinant individuals, mutant parent ‘JC007-2B’ and its same originated DH line population established that the mutation sites in *Bo3g002080* exhibited a complete consensus. *Bo3g002080*, homologous to *Arabidopsis MED4*, was identified as the candidate gene for *BoVl*. Expression analysis showed that *Bo3g002080* displayed a 2158.85-fold higher expression at albino stage than that in green leaf stage. Transcriptome analysis showed that related pathways of photosynthesis and chloroplast development were significantly enriched in the white sectors, and relevant DEGs involved in these pathways were almost down-regulated. Overall, our study provides a new gene resource for cultivar breeding in ornamental kale and contributes to uncovering the molecular genetic mechanism underlying the variegated leaf formation.

## 1. Introduction

Ornamental kale (*Brassica oleracea* var. *acephala*) is an ideal landscape plant suitable for flowerbeds and flower seas by virtue of its cold tolerance and attractive leaf coloration. It is becoming increasingly renowned for a wide range of color patterns in the inner leaves under lower temperatures, mainly including red, purple, pink and white. Currently, most research focuses on the color variation and the genetic mechanism of red and purple inner leaves [1,2,3,4,5], while little is known about the occurrence of variegated leaves in kale [6]. Leaf variegation refers to different-colored patches developed on the same leaf tissue, usually consisting of green and white (or yellow) sectors [7]. In our previous study, a variegated leaf kale showing green margins and white centers, which looks like a lotus and is of unique ornamental characteristics, was created by isolated microspore culture. Although the color transition of kale leaves from green to white could occur naturally, accompanied by some variegated leaves produced, this phenotype would fade away after a brief period. Therefore, such a distinctive and long-lasting green-white variegated leaf mutant is of great value for cultivar breeding and product application in ornamental kale, and provides a favorable tool for dissecting the formation mechanism underlying leaf variegation pattern.

For the variegated leaves, the green sectors possess morphologically normal chloroplasts, whereas the lighter colored sectors mostly contain aberrant chloroplasts, deficient in chlorophyll and carotenoid pigment contents [8]. Efforts have been made to explore the genetic model of variegated leaves since the early 20th century, while most of the traits in earlier research did not follow Mendelian inheritance, whose phenotypic formations were closely associated with plastid dysfunction. In recent years, with extensive investigations conducted into variegated leaf mutants of the model plant *Arabidopsis thaliana*, many nuclear genes modulating leaf variegation have been characterized. *VARIEGATED 2* and *VARIEGATED 3* determine the variegated leaves formation through regulating chloroplast development [9,10], while *IMMUTANS, MUTATOR*, *CLOROPLASTOS ALTERADOS*, *ATASE 2-DEFICIENT*, *GERANYLGERANYL DIPHOSPHATE SYNTHASE* (*GGPS*)*1* and *CRUMPLED LEAF* (*CRL*) control leaf variegation through regulating chlorophyll and biosynthesis [11,12,13,14,15,16].

Among these mutants, two representative mutants *immutans* (*im*) and *variegated 2* (*var2*) were characterized more fully and provided a theoretical basis for dissecting variegated leaf formation. The *im* mutant was first found by Rédei (1963), in which green sectors on the leaves contain normal cell wall and chloroplast, whereas the white sectors contain vacuolated plastids and abnormal lamellar structure [17,18]. Wu et al., (1999) mapped the recessive nuclear gene controlling this variegated leaf trait on the chromosome 4 of *Arabidopsis* [19]. IM protein serves as a plastid terminal oxidase regulating electron transport and redox, deficient in which would result in the formation of reactive oxygen, in turn leading to photo-oxidized plastids in the white sectors containing lower level of carotenoids and higher level of carotenoid precursor phytoene [11,17,19,20]. Another variegation-related mutant *var2* was isolated by Martínez-Zapater (1993), which has yellow-green variegated leaves that were susceptible to light and temperature, and was controlled by a pair of recessive nuclear genes [21,22]. The candidate gene *FtsH*, belonging to the AAA (ATPase associated with various cellular activities) ATPase superfamily, which were anchored on the transmembrane domain in thylakoids. Mutated *FtsH* led to abnormal thylakoid function, and affected the chloroplast development. Based on previous studies and these typical mutants, Yu et al., (2007) tentatively summarized seven types of molecular mechanisms underlying variegated leaf formation: genotype chimerism, transposon insertion, RNA silencing, plastome mutation, nuclear gene variation, mitochondrial genome mutation and plastid–nucleus incompatibility [23]. Moreover, three classic hypothesis models of variegated leaf formation have been proposed, namely, the photo-oxidation hypothesis, the FtsH complex threshold hypothesis and the chloroplast development and homeostasis hypothesis [23]. These results lay a solid foundation for uncovering the molecular mechanism of variegation formation throughout the plant kingdom.

The occurrence of leaf variegation in kale renders it with richer color patterns and ornamental characteristics, which would attract more interest, so it is necessary to explore the formation mechanism behind it for a better application. However, so far, there is no direct report on the molecular genetic mechanism of variegation formation in kale. In the present study, we characterized a novel variegated leaf mutant, the ‘JC007-2B’, appearing a peculiar green-white leaf variegation. Detailed morphological observation and physiological assays at different development stages revealed that chloroplast development and photosynthetic pigment contents were abnormal in the white sectors. Genetic analyses suggested that the leaf variegation was controlled by a dominant nuclear gene, *BoVl*. BSR, SSR and Indel markers were applied to conduct the fine mapping of the candidate gene. Sequence alteration with consensus detected in multiple variegated plants of two base substitutions and a base insertion on exon 2 of *Bo3g002080*, along with its distinctly differential high expression levels, further confirmed that *Bo3g002080* was the candidate gene for variegated leaf phenotype. *Bo3g002080* is homologous to *Arabidopsis MED4* encoding a hydroxyproline-rich glycoprotein family protein, but the function of *MED4* in affecting chloroplast development or determining pigment distribution has not been reported yet. To gain insight into the molecular mechanism underlying leaf variegation under the control of *BoVl*, we compared the transcriptomes of the green and white sectors in ‘JC007-2B’ and analyzed the DEGs, among which the related pathways of photosynthesis, chloroplast development and energy metabolism were significantly enriched in the white sectors, and relevant genes were almost down-regulated. These results contribute to uncovering the molecular mechanism of variegated leaf formation, and provides new insights for germplasm innovation in ornamental kale breeding.

## 2. Results

### 2.1. Characteristics of the Variegated Leaf Phenotype of ‘JC007-2B’

**Figure 1:**
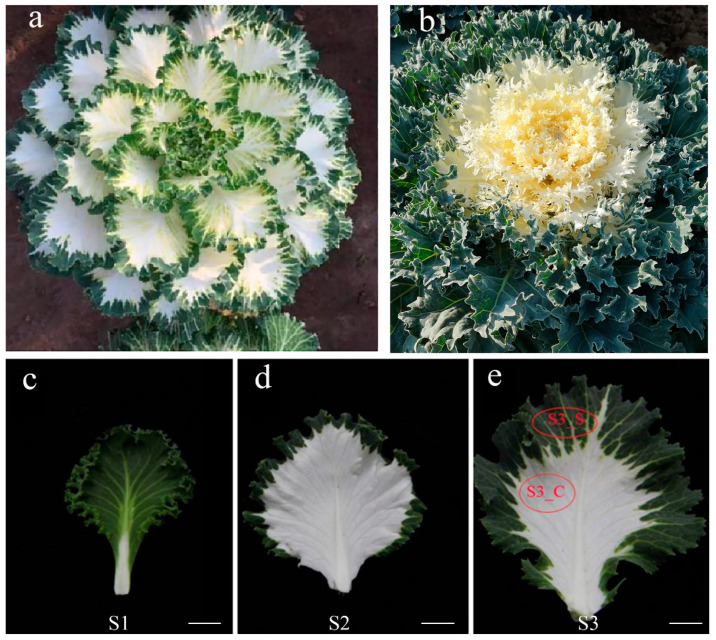
(**a**) The phenotype of the variegated leaf mutant ‘JC007-2B’; (**b**) The intermediate phenotype in common white-leaf kale; (**c**–**e**) variegated leaf patterns in ‘JC007-2B’ at different developmental stages; S1 (green leaf stage), leaf entirely green; S2 (albino stage), white appearing in the leaf center with a small green portion around leaf margin; S3 (variegated leaf stage), white leaf center and green leaf margin lasting during the whole ornamental stage; The green margin at S3 was denoted as S3_S, and the white center was denoted as S3_C. Bars = 1.75 cm.

The natural variegated leaf mutant ‘JC007-2B’ was initially obtained from the microspore cultured double haploid population of ‘Osaka red’. The leaves of the mutant began to turn color in response to low temperature, and thereafter, the white color of its inner leaves was gradually rendered with the decrease in environmental temperature. Along with the plant growth, the whole plant was featured by the variegated leaf phenotype with white leaf centers and green leaf margins (Figure 1a), which was long-lasting for the whole ornamental stage (Figure 1c–e). Unlike the intermediate phenotype in common white-leaf kale (Figure 1b), the variegated leaf phenotype of ‘JC007-2B’ would not disappear, which is of excellent ornamental value.

### 2.2. The Variegated Leaf of ‘JC007-2B’ Displayed Abnormal Pigment Accumulation

**Figure 2:**
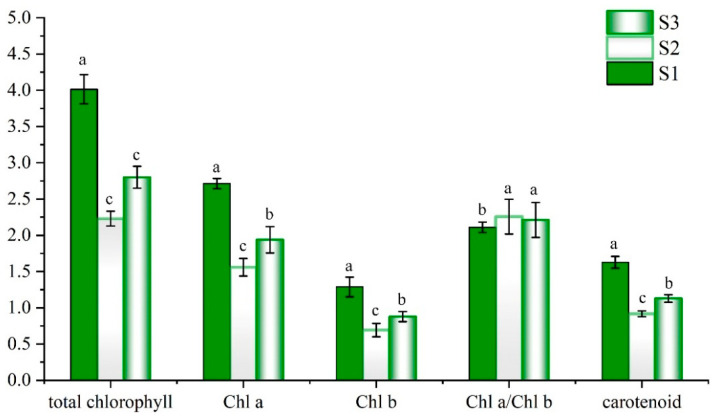
Determination of chlorophyll content, chlorophyll *a* content, chlorophyll *b* content, Chl *a*/Chl *b* ratio and carotenoid content of ‘JC007-2B’ at three different developmental stages. The unit of pigment content is mg/g.DW. The significance of the difference by SPSS. Different letters represent significant differences, *p* ≤ 0.05.

**Figure 3:**
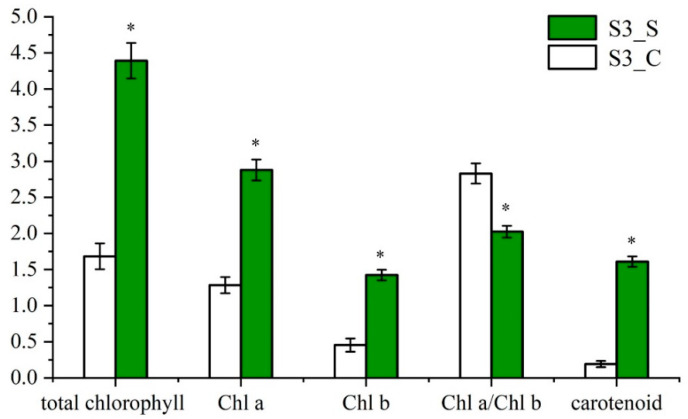
Determination of chlorophyll content, chlorophyll *a* content, chlorophyll *b* content, Chl *a*/Chl *b* ratio and carotenoid content of ‘JC007-2B’ in the S3_C and S3_S sections. The unit of pigment content is mg/g.DW. The significance of the difference by *t*-test, * represent significant differences, *p* ≤ 0.05.

To detect the physiological change in leaf color, we firstly measured total chlorophyll (Chl), chlorophyll *a* (Chl *a*), chlorophyll b (Chl *b*) and carotenoid (Car) content at different stages. As depicted in Figure 2, the total Chl, Chl *a*, Chl *b* and Car content reached the lowest at S2, whereafter it increased at S3, but the content was still lower than that at S1. The ratio of Chl *a*/Chl *b* was also calculated at three stages, which was higher at S2 and S3, indicating that the content of antenna pigments (Chl *b*) decreased to a level lower than that of the reaction center chlorophyll (Chl *a*). Subsequently, we measured Chl, Chl *a*, Chl *b* and Car content in the white and green region at S3, respectively. It turned out that the Chl, Chl *a*, Chl *b* and Car content in the white sectors at S3 were remarkably lower than that in the green leaves (Figure 3). This result suggested chlorophyll biosynthesis in the white region of leaves was disrupted during the plant growth.

### 2.3. Chloroplast Morphology Was Defective in the Variegated Leaf of ‘JC007-2B’

**Figure 4:**
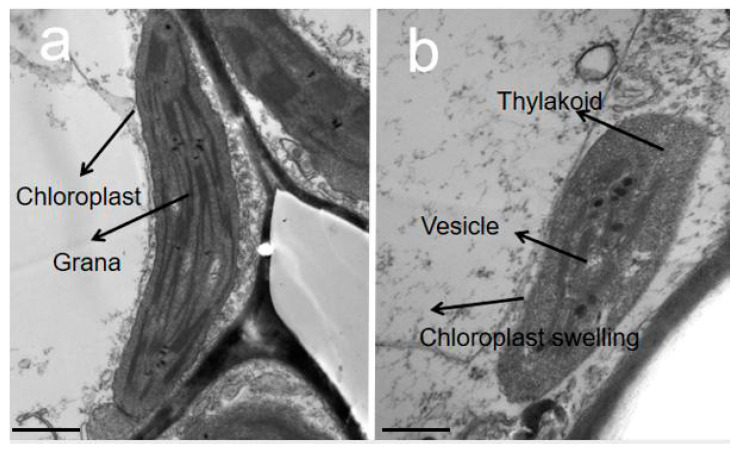
Ultrastructural observation of the chloroplast in the variegated leaf at S3. (**a**) Chloroplast ultrastructure in S3_S; Bars = 600 μm (**b**) Chloroplast ultrastructure in S3_C. Bars = 300 μm.

To examine if chlorophyll loss gave rise to the developmental abnormality in chloroplast, chloroplast ultrastructure in S3_C and S3_S sectors were inspected by TEM. The chloroplast and cell wall structures in the green sectors of S3 leaves were clear, the thylakoids and grana lamellae were well developed, and the latter were arranged systematically (Figure 4a). By contrast, the chloroplasts in the white sectors of S3 leaves contained osmiophilic granules, and there were fewer thylakoids and grana lamellae, as well as the membrane structures in chloroplast could not be clearly distinguished (Figure 4b). These results indicated that the chloroplast development of the white sections in variegated leaf was abnormal, which might affect the photosynthesis, in turn, causing the variegated leaf.

### 2.4. The Variegated Leaf Phenotype of ‘JC007-2B’ Was Controlled by a Pair of Dominant Nuclear Gene

For identifying the inheritance of variegated leaf trait, the ‘JC007-2B’ (variegated leaf) was crossed with the ‘BS’ (green leaf) to generate two F_1_ populations (Figure 1a, b). The phenotype of all F_1_ individuals exhibited variegated leaf trait, indicating the variegated leaf trait was controlled by dominant nuclear genes (Table 1). The F_2_ populations were obtained by selfing the F_1_ plants, while the BC_1_ populations were obtained by crossed with F_1_ populations and two parents. A total of 117 plants of F_2_ population were obtained, including 90 individuals with variegated leaves and 27 individuals with green leaves. The chi-square test showed that the segregation ratio (3.33: 1) of the mutant trait conformed to the Mendelian segregation ratio of 3:1 (χ^2^ = 0.17 < χ^2^_0.05,1_ = 3.84). In addition, a total of 11 plants of the 24 plants from BC_1_ population ((P1 × P2) × P2) possessed the variegated leaf trait, which obeyed the Mendelian segregation ratio of 1:1 (χ^2^ = 0.17 < χ^2^_0.05,1_ = 3.84). Collectively, these results demonstrated that the variegated leaf trait was controlled by the dominant nuclear gene, *BoVl*.

### 2.5. Primary Mapping of the BoVl Gene via BSR-Seq Analysis

**Figure 5:**
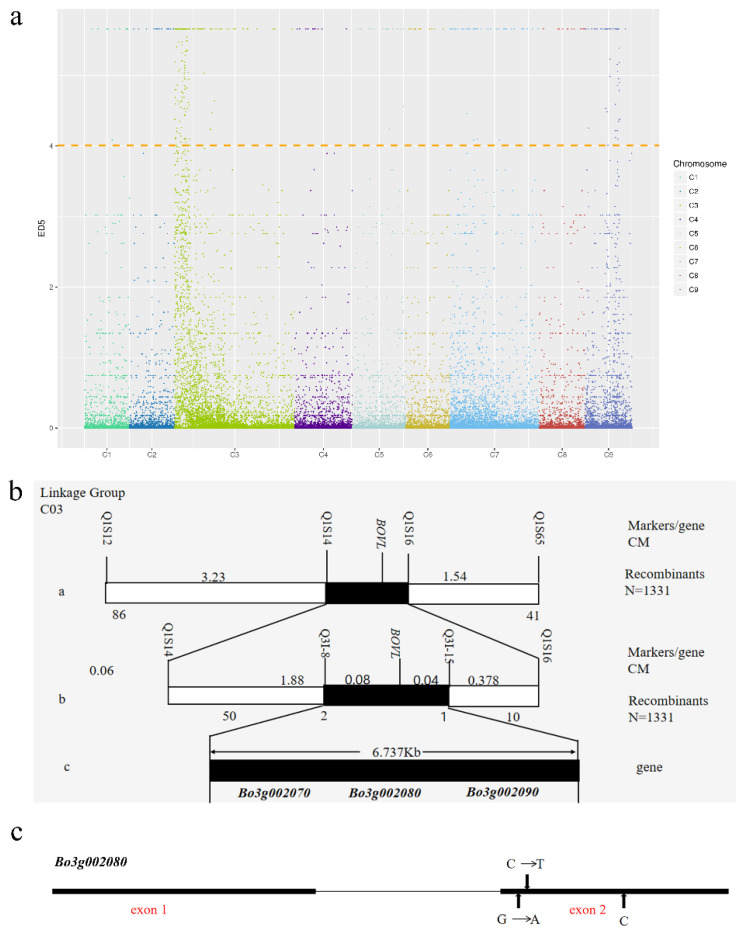
Genetic maps of the *BoVl* locus and analysis of the candidate gene on *Brassica oleracea* chromosome 9. (**a**) Distribution of ED^5^ values on chromosomes. Different colors in the panel indicate different SNV sites on different chromosomes; The width of each color in the x-axis represents the number of differential SNV loci in each chromosome; The ordinate represents the ED^5^ of each differential SNV locus; Threshold value of top 1% for horizontal line; (**b**) Physical maps of the variegated leaf gene, *BoVl*, in ornamental kale. The mutant gene *BoVl* is initially located on C03, and the mutant gene is located between markers Q1S14 and Q1S16. The physical distance was 56.67 Kb, including 17 candidate genes, and the genetic distance was 3.23 cM and 1.54 cM, respectively; Ultimately, the mutant gene *BoVl* was mapped to the interval between markers Q3I-8 and Q3I-15, whose physical distance is 6.74 Kb and contained three candidate genes; (**c**) Gene structure and the base change sites of *Bo3g002080*.

RNA of the variegated leaves and green leaves of F_2_ populations were applied to construct two pools, Var pool (variegated leaves) and CK pool (green leaves), and the two pools were sequenced to perform BSR-seq analysis. A total of 49,679,016 and 46,659,032 clean reads were acquired from Var pool and CK pool, and the data were used for further analysis (Table 2). The Q20 of the two pools were 97.80% and 97.75%, and the Q30 of the two pools were 94.16% and 94.06%, respectively. The SNV analysis were performed to calculate the ED^5^ value. According to the top 1% SNV locus, *BoVl* was mapped to three regions on C03 and C09 (Table 3, Figure 5a).

### 2.6. Fine Mapping of the BoVl Using Genome Sequence Data

Based on the preliminary mapping result via BSR-seq, we developed 90 SSR markers distributed on the two chromosomes to verify the accuracy of preliminary positioning results and, further, to narrow the location interval and refine the position of *BoVl*. Among the markers, seven SSR markers exhibited significant polymorphism between two parents. Two hundred green leaf F_2_ individuals were selected to confirm whether the markers were linked to *BoVl*. As a result, Q1S12 and Q1S65, which were around the first region on C03, were linked with *BoVl*. Then, 1331 green leaf individuals were selected for fine mapping, and the results showed that Q1S65 and Q1S12 were linked to *BoVl* at a genetic distance of 1.54 cM and 3.23 cM, respectively (Figure 5b). SSR and Indel markers were developed within the mapping region according to the genome sequence from database and re-sequencing data of two parents. Four markers (Q1S14, Q3I-8, Q3I-15 and Q1S16) were linked to *BoVl*. Among them, Q1S14 and Q3I-8 were on the same side as Q1S12, at a distance of 1.96 and 0.08 cM from *BoVl*, respectively, while Q3I-15 and Q1S16 were on the other side at a distance of 0.04 and 0.42 cM from *BoVl*. The physical distance between the two closest markers was 6.74 Kb, and the region contained three genes, namely *Bo3g002070*, *Bo3g002080* and *Bo3g002090*.

### 2.7. Analysis of the Candidate Genes for BoVl

Based on the results above, the function of homologous *Arabidopsis* genes to the three candidate genes was referred and analyzed. *Bo3g002070*, encoding a ubiquitin-protein ligase, contains a HECT domain and is homologous to the *AT5G02840* in *Arabidopsis*. *AT5G02840* encodes REV4, a homolog of the circadian rhythm regulator RVE8. The rev4rev6rev8 triple mutant showed an extremely long circadian, and the evening-phased clock genes expression level were reduced. The gene also involved in heat shock response and cold response. *Bo3g002080* encodes a hydroxyproline-rich glycoprotein family protein and is homologous to *AT5G02850* (*AtMED4*), a member of a conserved multi-subunit complex (MEDIATOR) that links transcription factors and RNA polymerase II, and promotes transcription. *Bo3g002090* encodes a ribosomal protein L4 family protein and is homologous to *AT5G02870*, a member of ribosomal protein. The gene had been reported to be involved in leaf development, plant growth and stress tolerance. Yet, no study has reported that any of these three genes are involved in the formation of variegated leaves.

Primers were designed according to the genome sequence and were used to clone the full gene length of the three genes in the mapping parents. The results showed that the gene sequences of *Bo3g002070* and *Bo3g002090* of ‘JC007-2B’ were different to those of ‘BS’, but the differences were all the same to those of the reference genome sequence from non-variegated phenotype cabbage (Figures S1 and S2). The full length of *Bo3g002080* is 972 bp, which contains two exons and one intron. A sequence alignment of two mapping parents showed that there existed two base substitutions and a base insertion on exon 2, and the gene sequence of ‘BS’ was same as those of the reference genome sequence (Figure 5c and Figure S3a). The gene sequence of *Bo3g002080* of ‘JC007-2B’ can translate 679 amino acids (aa). The conserved domain was located on the 6th–143rd aa, and the base substitution on the 395th (G→A) changed the 132nd aa from Arg to Lys. In addition, the base insertion (C) on exon 2 led to a frameshift mutation resulting in only 194 aa being translated (Figure S3b). These results revealed that *Bo3g002080* is likely to be the candidate gene for *BoVl*.

### 2.8. Bo3g002080 Was the Putative Candidate Gene for BoVl

To further verify the hypothesis, the genome sequence of *Bo3g002080* was amplified from five recombinant individuals between the two most closely linked markers: Q3I-8 and Q3I-15. Sequence alignment results showed that the fragment lengths of all recombinant plants in F_2_ population were consistent with that of ‘BS’ (Figure S4a). We also cloned the *Bo3g002080* gene sequence from the non-variegated leaf phenotype kale plants and the DH line plants derived from the same origin as ‘JC007-2B’; the results showed that the gene sequences from non-variegated leaf phenotype kale were identical with that of ‘BS’, while the gene sequences of the DH line plants were same as that of ‘JC007-2B’ (Figure S4b). These results further confirmed that the mutation locus in *Bo3g002080* shared a consistency in variegated leaf kale lines, which was the putative candidate gene for the *BoVl* gene conferring the variegated leaf phenotype.

**Figure 6:**
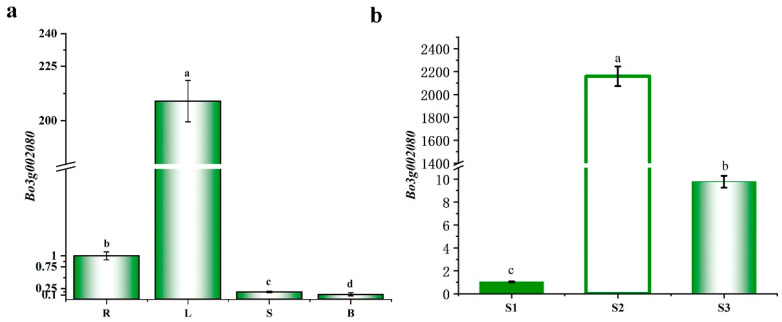
Expression pattern of *Bo3g002080*. (**a**) The expression level of *Bo3g002080* in different tissues from ‘JC007-2B’; (**b**) The expression level of *Bo3g002080* at different developmental stages of ‘JC007-2B’; Different letters represent significant differences, *p* ≤ 0.05; R: root: L: leaf; S: stem; B: bud; Different letters represent significant differences, *p* ≤ 0.05.

In addition, the expression pattern of *Bo3g002080* was detected in different tissues from ‘JC007-2B’, including roots, leaves, stems and pods. The results showed that *Bo3g002080* were expressed at highest level in the leaves, which was 209 time higher than that in roots (Figure 6a). We further examined the expression level of *Bo3g002080* at three development stages. As shown in Figure 6b, the expression level of *Bo3g002080* in ‘JC007-2B’ at S2 reached the highest, and the expression level was 2158.85-fold higher than that at S1. Collectively, these results strongly reflected that *Bo3g002080* was the most likely candidate gene for *BoVl*.

### 2.9. Hydrophobicity and Protein Structure Analyses

The conserved domain of *Bo3g002080* encoded protein in ‘BS’ and ‘JC007-2B’ was analyzed by PHMMER. It showed that the conserved domain, Vitamin-D-receptor-interacting Mediator subunit 4, was located between the 6th–143rd aa of ‘JC007-2B’, in which the mutation site in the 132nd aa happened to be located within the conserved domain (Figure S5a). The protein hydrophobicity was analyzed between ‘JC007-2B’ and ‘BS’ by ProtScale, which turned to hydrophilic protein in ‘JC007-2B’ (Figure S5b). The secondary structure of ‘JC007-2B’ and ‘BS’ was predicted by SOPMA software, and both proteins were composed of α-helix, β-sheet, random coil and extended chain. However, the protein in ‘JC007-2B’ had shorter random coils and longer helixes than that in ‘BS’ (Figure S5c). The results of tertiary structure analysis were similar to those of secondary structure analysis (Figure S5d). We further constructed a phylogenetic tree to analyze the evolutionary relationship between the two proteins and the proteins from other species. The results showed that the two proteins were closely related to the proteins from *Arabidopsis*. It also has a close genetic relationship with cruciferous plants such as white mustard, cauliflower, *Brassica napus*, purslane, Chinese cabbage and turnip protein (Figure S5e).

### 2.10. Comparative Transcriptome Analysis between S3_S and S3_C

#### 2.10.1. The GO and KEGG Analysis of S3_S vs. S3_C

**Figure 7:**
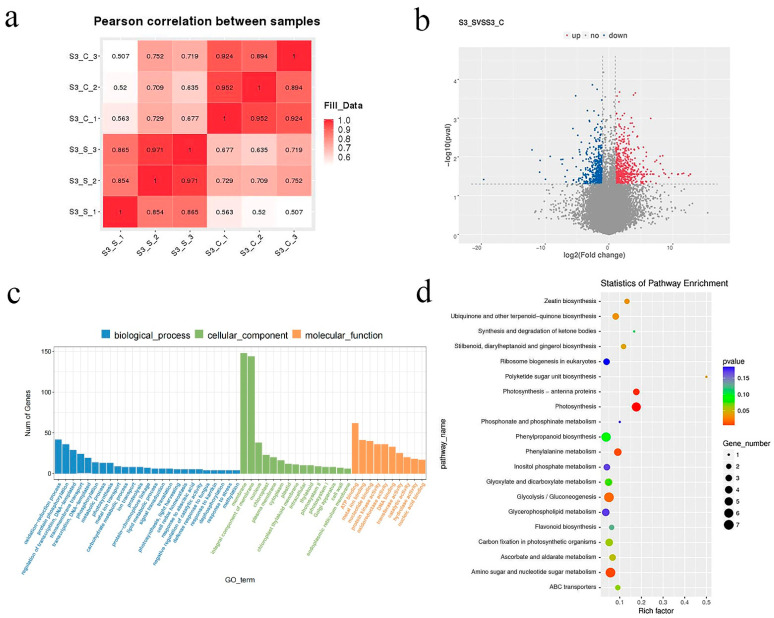
GO and KEGG analysis of the DEGs in variegated leaf ornamental kale by RNA-Seq. (**a**) Pearson correlation between samples; (**b**) Volcano plots of differential expressed genes; (**c**) Analysis of GO enrichments for S3_S vs. S3_C; (**d**) Enriched KEGG pathways for S3_S vs. S3_C (*p* value ≤ 0.05).

To probe the potential molecular mechanism of the formation of variegated leaf phenotype of the ‘JC007-2B’, comparative transcriptome analysis was performed between the green margin (S3_S) and white center (S3_C) of ‘JC007-2B’. A total of 291.18 million valid reads were generated (Table S1), from which approximately 88% (88.41–89.48%) of those were mapped to the *B. oleracea* genomes for expression analysis (Table S2, Figure 7a). A total of 1033 DEGs were identified between the white centers and the green margins, and 596 of them were upregulated in the S3_S (Figure 7b), whereafter the DEGs were assigned to 108 GO terms in three categories (Figure 7c). The terms associated with the chloroplast, plastid development, and photosynthesis/photosystem were sorted out and were aligned to the reference canonical pathways in the KEGG database (Figure 7c). The KEGG analysis displayed that DEGs were enriched significantly in nine pathways, with the most differentially expressed genes in ‘Amino sugar and nucleotide sugar metabolism’ (7), ‘Glycolysis/Gluconeogenesis’ (7) and ‘Photosynthesis’ (6), as well as the DEGs related to chloroplast development were up-regulated (Figure 7d).

#### 2.10.2. Relevant DEGs of Leaf Variegation and the Validation by qRT-PCR

**Figure 8:**
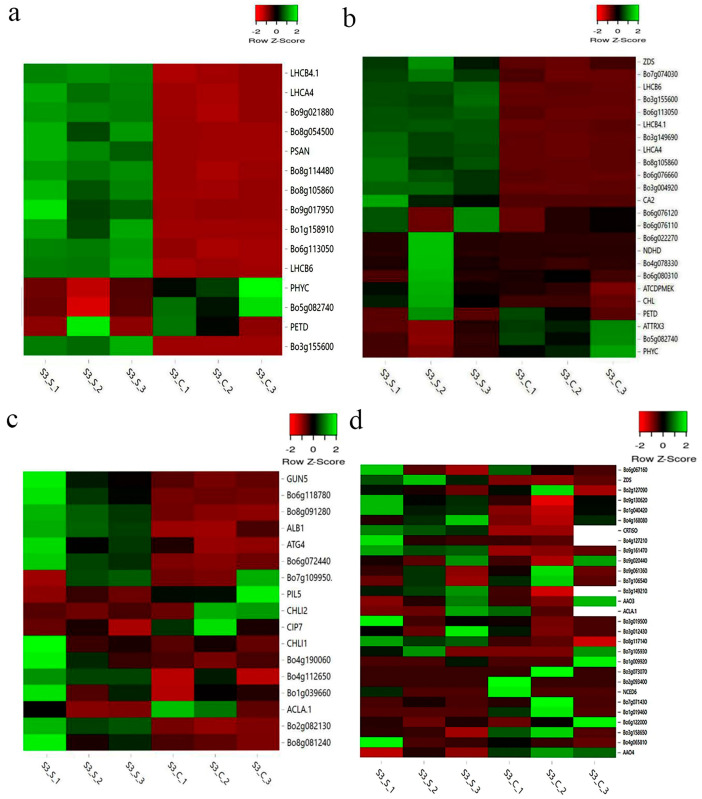
Comparative analysis of the DEGs or genes related to the phenotype formation. (**a**) Heatmap showing the expression profiles of DEGs related to photosynthesis; (**b**) Heatmap showing the expression profiles of DEGs related to chloroplast tissue; (**c**) Heatmap showing the expression patterns of genes related to chlorophyll biosynthetic process; (**d**) Heat map showing the expression patterns of genes related to carotenoid biosynthesis.

**Figure 9:**
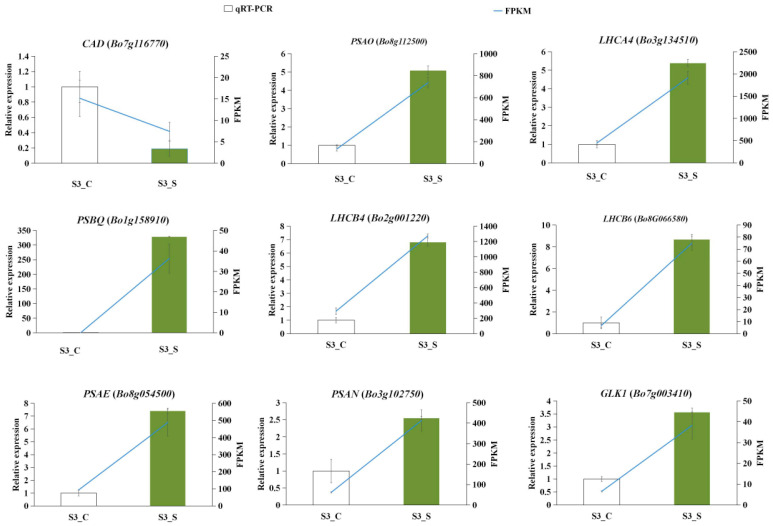
The validation and expression analysis of photosynthesis-related genes using qRT-PCR.

Considering that chloroplast is a crucial organelle containing chlorophyll for photosynthesis and is composed of three parts: chloroplast envelope, stroma and thylakoid, we put emphases on the photosynthesis and chloroplast-related pathways that were functionally closely associated with variegated leaf formation. Based on the GO and KEGG analysis, we concluded 15 genes involved in photosynthesis-antenna proteins and photosynthesis were denoted as DEGs, including *PsaE* (*Bo8g054500*), *PsaN* (*Bo9g017950* and *Bo3g102750*), *PsbO* (*Bo9g021880*), *PsbP* (*Bo8g114480*), *PsbQ* (*Bo1g158910*), *CP47* (*Bo3g155600*), *Cab* (*Bo5g082740*), *2Fe-2S* (*Bo6g113050*), *CP24* (*Bo8g105860*), *LHCA4* (*Bo3g134510*), *LHCB4.1* (*Bo2g001220*), *LHCB6* (*Bo8g066580*), *PETD* (*Bo8g030510*) and *PHYC* (*Bo6g056310*), most of which were expressed higher in the green margins (Figure 8a). Twenty-three DEGs mainly involved in chloroplast structures and activities were detected and so too were eleven of the DEGs: *CP47* (*Bo3g155600*), *Cab* (*Bo5g082740*), *2Fe-2S* (*Bo6g113050*), *CP24* (*Bo8g105860*), *CA2* (*Bo2g012160*), *CHL* (*Bo3g113850*), *LHCA4* (*Bo3g134510*), *LHCB4.1* (*Bo2g001220*), *LHCB6* (*Bo8g066580*), *NDHD* (*Bo9g097270*) and *PETD* (*Bo8g030510*) were involved in the formation of chloroplast thylakoid membrane. More than half of these DEGs involved in chloroplast tissue were expressed higher in the green margins (Figure 8b). We also focused on the genes related to chlorophyll biosynthesis and carotenoid biosynthesis in accordance with the lower level of photosynthetic pigments content in ‘JC007-2B’ white sectors at S3. There were 17 and 28 genes related to chlorophyll and carotenoid biosynthesis, respectively (Figure 8c, d). Among these genes, *ZDS* (*Bo5g146930*) was identified as the DEG. Combining the function annotations of these DEGs with the abnormal chloroplast development and the reduced photosynthetic pigment contents in S3_C (Figure 2, Figure 3 and Figure 4), it was conjectured that the variegated leaf phenotype arising via *BoVl* (*Bo3g002080*) was closely related to the carotenoid biosynthesis, chloroplast development and photosynthesis pathways. In order to verify the reliability of these DEGs, the expression patterns of nine photosynthesis-related DEGs and another nine randomly selected genes were examined by qRT-PCR. The gene expression patterns showed similar trends consistent with that observed by RNA-seq analyses (Figure 9 and Figure S6).

## 3. Discussion

Leaf variegation can attach rare appearances and unusual esthetic value to ornamental plants. In recent years, ornamental kale has been widely introduced as a decorative landscape plant around the world because of its attractive leaf color pattern and low-temperature tolerance. In this study, the variegated leaf mutant ‘JC007-2B’ (Figure 1a) and the *BoVl* loci are of great value for breeding *B. oleracea* new varieties. The variegated leaves with white centers and green edges in ‘JC007-2B’ were gradually developed along with the daily mean temperature dropping to roughly 12 °C. Notably, as a novel type of ornamental phenotype in kale, the variegated leaf phenotype in ‘JC007-2B’ would not disappear as the plant growth, which is distinctive from the intermediate phenotype in common white-leaf kale (Figure 1b). Such kinds of mutants in which variegated leaves can be stably inherited and preserved make it possible for artificial selection and the development of new ornamental cultivars. Accordingly, the coloration mechanisms of variegated leaves mutants aroused more interests and have been studied at multiple levels in multiple species [24,25,26,27].

The color variations in green-white variegated leaves were almost accompanied by prominent characteristics of the alterations in chlorophyll and carotenoid contents as well as the abnormality in chloroplast development of the white sectors. Leaf-variegated mutants are thereby regarded as ideal materials for investigating the chloroplast function. Likewise, we determined total chlorophyll, chlorophyll *a*, chlorophyll *b* and carotenoid contents at three development stages, respectively. It was found that these pigment contents reached the maximum at green leaf stage (S1) and the lowest at albino stage (S2), as well as significantly lower in the white sectors (S3_C) than the green sectors (S3_S) at variegated leaf stage (Figure 2 and Figure 3). By observing the ultrastructure of chloroplasts in the green and white areas of variegated leaf, there were abnormal chloroplasts about to collapse in the S3_C rather than structurally clear chloroplasts with highly stacked thylakoids in the S3_S (Figure 4). Similarly, aberrant chloroplasts with loose grana lamellae and indistinct thylakoid membrane structure were also observed in other green-white variegated leaf mutants including *Csvl* of cucumber (*Cucumis sativus* L.), *Hvcmf7* of barley (*Hordeum vulgare*) and white sectors in variegated leaves of milky stripe fig (*Ficus microcarpa*) [28,29,30]. Furthermore, chloroplast is the main place for plant energy transformation, and most of the photosynthetic pigments exist in the thylakoid membrane absorbing light energy through the photosystem for photosynthesis [11,19,20]. The white centers in S3 showed an increase in Chl *a*/Chl *b* ratio opposite to the decreasing trend in each single pigment, which suggested the Chl *b* contents declined to a greater extent. Chl *a*/Chl *b* ratio can reflect the plant utilization rate of light energy, and we speculate that the white leaves might be subjected to a certain degree of light stress. The Chl *b* is only present in the antenna complex and plays an indispensable role in affecting photosynthetic performance [31]. Accordingly, the DEGs involved in photosynthesis-antenna proteins and photosynthesis were identified, most of which were up-regulated and expressed in the green sectors (S3_S). Based on the aforementioned findings, we speculated that the abnormal photosynthetic pigment contents and damaged chloroplast structure in the white centers led to the green-white appearance of variegated leaves in ‘JC007-2B’ at the physiological level.

Currently, relevant studies of variegated leaf formation in other species besides *Arabidopsis* mainly focused on unravelling potential functional pathways and mutant analysis, whereas the genetic models accounting for the variegated leaf phenotype have not been fully identified. In this study, two mapping parents, variegated leaf ornamental kale ‘JC007-2B’ and green-leaf inbred line ‘BS’ of *Brassica oleracea*, were used to map the candidate gene. It was proved that the variegated leaf trait was controlled by a dominant nuclear gene. Genetic mapping of the *BoVl* locus was carried out by BSR seq and molecular markers, which was located in a 6.74 Kb interval of C03, flanked by Q3I-8 and Q3I-15. A total of three genes were detected in the interval. Through sequence alignment, two non-synonymous base substitutions and a base insertion were found in the exon 2 of *Bo3g002080* (Figure 5), which occurs in the 132^nd^ amino acid, within the Vitamin-D-receptor-interacting Mediator subunit 4 conserved domain (Figure S5a). The nucleotide mutation at the *BoVl* locus shared a consistency among the variegated leaf kale lines, indicating that *Bo3g002080* was the most likely candidate gene for *BoVl* (Figure S4).

*Bo3g002080* is homologous to *Arabidopsis MED4* encoding a hydroxyproline-rich glycoprotein family protein, which is a component of the middle module in the mediator, wherein seven proteins that are conserved across evolutionary time and eukaryotes constitute this module [32,33,34,35]. In yeast, MED4 serves as a hub and interacts with all of the other proteins in the middle module [32]. Studies showed that the *Arabidopsis MED4* mutations exhibit the embryonic mortality phenotype, and MED4 can interact with three types of RNA polymerases [36]. Up to now, there are few studies on the function of MED4, and whether MED4 affects chloroplast development remains to be studied. In the future, additional experiments, such as transgenic complementation test, CRISPR/Cas9 and RNAi, are needed to uncover the role of *BoVl* (*Bo3g002080*) in leaf variegation.

Analysis of the differentially expressed genes and related gene regulatory network in variegated leaf formation will facilitate our understanding of the molecular genetic mechanism of kale color pattern and decipher the potential function of *BoVl*. To date, a total of three classic hypotheses were proposed for explaining the intricate mechanism underlying leaf variegation. The first one is that the mutation of chlorophyll and carotenoid biosynthesis or related genes that lead to changes in pigments content, such as *MUTATOR*, *CLOROPLASTOS ALTERADOS*, *GGPS1* and *CRL* mutants [9,11,12,13,14,15]; the second hypotheses is that the mutation of chloroplast developmental genes that lead to anormal chloroplast development therefore indirectly affects pigments synthesis, such as *VARIEGATED 2* (*var2*) and *VARIEGATED 3* (*var3*) mutants [9,10]; the third possibility is that photooxidation, which results from gene deletion or mutation during photosynthetic electron transport, will affect the equilibrium between electron transport and plastid quinone’s redox reaction, leading to defects in chloroplast development, similar to those seen in *im* mutants [11,17]. Likewise, we also observed abnormal chloroplast development and decreased photosynthetic pigments content in the white sector of variegated leaves of ‘JC007-2B’. On the basis of these classic theories and the physiological assays, we put emphases on the photosynthesis, pigments biosynthesis and chloroplast-related pathways and DEGs in the comparative transcriptome analysis. Relevant DEGs related to carotenoid biosynthesis, chloroplast, thylakoid membrane, and photosynthesis were identified between the green margins (S3_S) and the white centers (S3_C) (Figure 7 and Figure 8). Thus, it was speculated that the blockage of carotenoid and chlorophyll biosynthesis resulted in the abnormality of chloroplast development, which ultimately induced the formation of variegated leaves.

**Figure 10:**
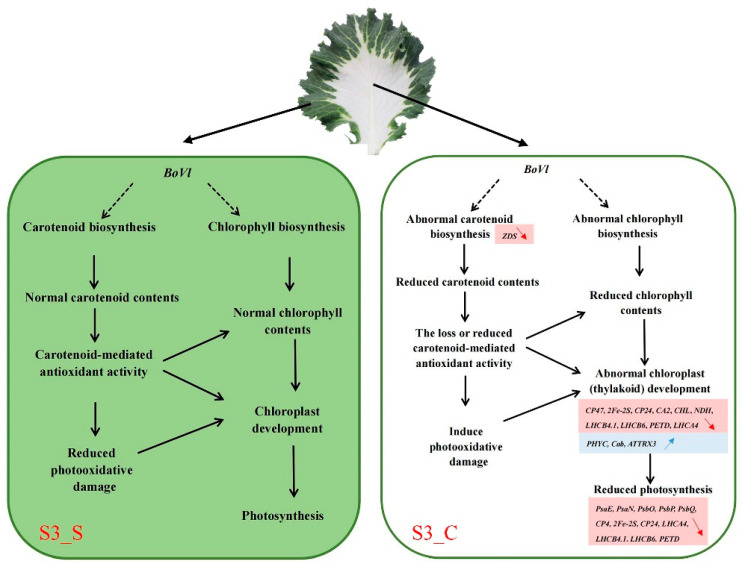
The proposed possible formation pattern of leaf variegation of ‘JC007-2B’.

Among these DEGs, we only found a gene, *ZDS*, encoding a ζ-carotene desaturase in carotenoid biosynthesis. Carotenoids as a class of natural pigments, were involved in a variety of physiological processes, including coloration, photoprotection, biosynthesis of abscisic acid, and chloroplast biogenesis. In addition, carotenoids play an important role in photoprotection by protecting plants from these oxidative damages [37]. The *Arabidopsis SPC1/ZDS* gene mutation resulted in damaged carotenoid biosynthesis, decreased chlorophyll content, and abnormal chloroplast structure, with a bleached phenotype of leaves. Similarly to the *Arabidopsis im* mutants, they were all subjected to different degrees of photooxidation [20,38]. Therefore, we speculated that the blockage of carotenoid biosynthesis and the decrease in contents might occur in the white region, which in turn could lead to a certain degree of photooxidation damage and abnormal chlorophyll synthesis, thereafter inducing abnormal chloroplast development. In addition, the expression of most DEGs involved in chloroplast thylakoid development and photosynthesis was decreased in S3_C. Gathering up these threads, a putative formation pattern of leaf variegation of ‘JC007-2B’ was proposed (Figure 10), which could contribute to the understanding of potential genetic mechanisms and the regulatory network of *BoVl* in leaf variegation of ornamental kale.

## 4. Materials and Methods

### 4.1. Plant Materials

The double-haploid ‘JC007-2B’ kale line was grown in the greenhouses at Shenyang Agricultural University (Shenyang, China). The kale exhibited a variegated leaf phenotype with white centers and green margins. Variegated phenotype during plant development were as follows: S1, leaves are green; S2, white appears at the leaf center, although the margins remain green when the plant undergoes a period of chilling temperature; and S3, the leaf center is white, while the margins are green (Figure 1). To investigate the genetic mechanism of variegated leaf formation, we crossed ‘JC007-2B’ with a *Brassica oleracea* inbred line ‘BS’ to produce F_1_. The F_1_ populations were selfed to generate F_2_ populations, and they were backcrossed with two mapping parents to produce BC_1_ lines, respectively. A total of 50 variegated leaf individuals and 20 green leaf individuals of F_2_ populations were used for BSR-seq analysis and then another 1331 green leaf individuals from F_2_ population were planted to fine map the *BoVl* gene. All populations were grown on land at Shenyang Agriculture University, Shenyang, China.

The leaf color pattern was determined by visual inspection when the populations underwent a period of chilling temperature. Chi-square test was used for verifying the segregation rate.

### 4.2. Measurement of Chlorophyll and Carotenoid Content

Green and white sectors in variegated leaves were selected for the measurement of chlorophyll and carotenoid levels. Dry weight samples (20 mg) were soaked in 10 mL of 96% ethanol solution (*v*/*v*), at 25 °C, for 24 h. The samples were centrifuged for 30 s every 12 h [39]. The absorbance was measured three times each at wavelengths of 649, 665, and 470 nm on an ultraviolet spectrophotometer (T6 New Century; Persee, Beijing, China). Total chlorophyll, chlorophyll *a*, chlorophyll *b*, and carotenoid contents were calculated using the following formulae:

Chlorophyll *a* content = 13.95 A_665nm_ − 6.88 A_649nm_

Chlorophyll *b* content = 24.96 A_649nm_ − 7.32 A_665nm_

$$\mathrm{Carotenoid content}\;=\;\frac{1000\times A_{470\mathrm{nm}}-2.05\times \text{chlorophyll}\;a\;\text{content}-114.8\times \text{chlorophyll }b\;\text{content}}{245}$$

### 4.3. Transmission Electron Microscopy

Leaves at three stages were cut into 1 mm^2^ sections, fixed in glutaraldehyde solution (2.5% glutaraldehyde, 0.1 M Na_2_HPO_4_, 0.1 M NaH_2_PO_4_ [pH 7.0]), washed in 0.1 M phosphate buffer, fixed in 1% OsO_4_ in the same phosphate buffer, and dehydrated in a graded series of acetone before embedding and polymerization in Epon 812. After ultrathin sectioning (LKB2088 type ultramicrotome; LKB Co., Bromma, Sweden), the samples were stained with uranyl acetate and lead citrate solutions and observed under a transmission electron microscope (H-7650; Hitachi, Tokyo, Japan). Images were acquired at 30,000× magnification [40].

### 4.4. BSR-Seq Analysis

Fifty variegated leaf individuals and fifty green leaf individuals of F_2_ populations were selected to construct two bulks, Var and CK, for BSR-seq analysis. Total RNA of each sample was extracted using TRIzol Reagent (Invitrogen), and were quantified and qualified by Agilent 2100 Bioanalyzer (Agilent Technologies, Palo Alto, CA, USA), NanoDrop (Thermo Fisher Scientific Inc.) and 1% agrose gel. The RNA with RIN value above 7 from fifty individual variegated leaves and fifty green leaves was mixed to construct two libraries, respectively. The next-generation sequencing library preparations were constructed according to the manufacturer’s protocol (NEBNext^®^ Ultra™ RNA Library Prep Kit for Illumina^®^). NEBNext Poly(A) mRNA Magnetic Isolation Module (NEB) was used to perform the poly(A) mRNA isolation. The mRNA fragmentation and priming was performed using NEBNext First Strand Synthesis Reaction Buffer and NEBNext Random Primers. First-strand cDNA and second-strand cDNA were synthesized using ProtoScript II Reverse Transcriptase and Second Strand Synthesis Enzyme Mix, respectively. Two libraries with different indices were multiplexed and loaded on an Illumina HiSeq X Ten instrument according to manufacturer’s instructions (Illumina, San Diego, CA, USA). The sequences were processed and analyzed by GENEWIZ (Nanjing, Jiangsu, China).

Trimmomatic (v0.30) was used to obtain high quality clean data. Then, clean data were aligned to reference genome (EnsemblPlant, *B. oleracea*, v2.1) via software Hisat2 (v2.0.1). HTSeq (v0.6.1) estimated gene and isoform expression levels from the paired-end clean data. DESeq Bioconductor package, a model based on the negative binomial distribution, helps us to analysis differential expression genes (DEGs). Additionally, the threshold was set as log_2_ (fold change) > 1 and statistical significance (*p* < 0.05). Samtools v0.1.18 with command mpileup and Bcftools v0.1.19 were used to do SNV calling. Additionally, ED value was calculated based on an mpileup file, which generated by samtools v0.1.18.

### 4.5. Genomic DNA Extraction, PCR and Molecular Marker Development for Fine Mapping

Young leaf samples of green leaf individuals of F_2_ populations and two parents were collected and frozen in liquid nitrogen. Genome DNAs were extracted from leaves from modified cetyltrimethylammonium bromide (CTAB) method [40]. The SSR loci were detected via SSR Hunter based on the *Brassica oleracea* genome, while the Indel loci were identified according to the results of genome re-sequencing and BSR-seq analysis. Both two types of markers were designed by Primer Premier 5.0. A total of 77 SSR markers were used to confirm the mapping region, then 15 Indel markers were used to narrow the region. Map distances were calculated referring to Kosambi’s (2011) mapping function [41].

The PCR reaction contains ~80 ng genome DNA, 0.5 μM of each primer, 200 μM dNTPs, 1× reaction buffer and 0.5 U Taq DNA polymerase (Tiangen, Beijing, China). The PCR program was as follows: 95 °C for 5 min, 35 cycles of 95 °C for 15 s, 58 °C for 30 s, 72 °C for 30 s, and 72 °C for 5 min. The PCR products were separated on a 6% polyacrylamide gel by electrophoresis, and electrophoresis at 200 V for 1.5 h. The gels were stained in 0.1% AgNO_3_ solution and then were transferred into a developing solution (1.5% sodium hydroxide, 0.4% formaldehyde).

### 4.6. Gene Annotation and Candidate Gene Identification

Candidate gene prediction was based on the *Brassica oleracea* genome database (ftp://ftp.ensemblgenomes.org/pub/release-38/plants/genbank/brassica_oleracea, accessed on 1 October 2022). The functions of the genes in the interval were analyzed using the BLASTP tool from TAIR. The gene and promoter sequence of the genes were amplified from two parents genomic DNA with PrimeSTAR^®^vMax DNA Polymerase (TAKARA, Dalian, China). All primers used for sequencing are listed in Supplementary Materials Table S3. The PCR products were purified using the Gel Extraction Kit (CWBIO, Beijing, China), introduced into the PMD 18-T Vector (Takara, Dalian, China), and transformed into TOP10 competent cells (CWBIO). The recombinant plasmids were sequenced by Genewiz (Tianjin, China) and sequences were aligned using DNAMAN 6 (https://www.lynnon.com/, accessed on 1 October 2022).

### 4.7. Phylogenetic Analysis and Alignment of BoVl and Its Homologous Proteins

The amino acid sequences of *BoMED4* and its homologous protein in different species were downloaded from the NCBI database. Protein sequence alignments were performed with Clustal W, and a neighbor-joining tree was constructed with 1000 replications. The proteins sequences used in the neighbor-joining tree were from the following species: the amino acid sequence, isoelectric point (PI) and molecular weight (MW) of the gene protein were estimated by Edit-seq; three candidate genes were sequenced by DNAman; the conserved domains of candidate genes were analyzed using Hmmer (http://plants.ensembl.org/hmmer/index.html/, accessed on 1 October 2022). We used: smart (http://smart.embl-heidelberg.de/, accessed on 1 October 2022) to predict the amino acid sequence of the candidate gene and the conserved domain of the candidate gene; sopma (http://npsa-pbil.ibcp.fr/cgi-bin/npsa_automat.pl, accessed on 1 October 2022) to predict the secondary structure of candidate genes; and Protscale (https://web.expasy.org/protscale/, accessed on 1 October 2022) to hydrophobicity prediction of candidate genes. The phylogenetic tree of reference genes *Bo3g002080*, ‘BS’ and ‘JC007-2B’ in kale and other species was constructed using MEGA11.

### 4.8. RNA-Seq Analysis

Total RNA was extracted using a Total RNA Purification kit (LC Science, Houston, TX, USA; TRK1001) according to the manufacturer’s protocol. RNA quantity and purity were evaluated using a Bioanalyzer 2100 and RNA 6000 Nano LabChip kit (Agilent Technologies, Santa Clara, CA, USA), with an RNA integrity number > 7.0. Poly(A) mRNA was isolated from 10 μg RNA using the poly-T oligo method (Invitrogen, Carlsbad, CA, USA). Cleaved RNA fragments were used to generate a cDNA library using an mRNA-Seq Sample Preparation kit (Illumina, San Diego, CA, USA) according to the manufacturer’s protocol. Samples from S3 (leaf margin and center) with three independent biological replicates were used to construct 6 cDNA libraries designated as S3_S_1, S3_S_2, S3_S_3, S3_C_1, S3_C_2, and S3_C_3. Paired-end sequencing was carried out using the Illumina HiSeq 4000 system (LC Sciences, Hangzhou, China) according to the manufacturer’s protocol. After removing reads of low quality, those that remained were mapped to the *B. oleracea* reference genome (ftp://ftp.ensemblgenomes.org/pub/release-38/plants/genbank/brassica_oleracea/, accessed on 1 October 2022) using the HISAT package, allowing for a maximum of two mismatches and multiple alignments per read (up to 20 by default).

Mapped reads of each sample were assembled using StringTie. The final transcriptome was generated by merging all transcriptomes using Perl scripts. mRNA expression levels were calculated by the fragments per kilobase million (FPKM) method using StringTie (https://ccb.jhu.edu/software/stringtie/, accessed on 1 October 2022), whereas differentially expressed mRNAs were identified based on log_2_ (fold change) > 1 and statistical significance (*p* < 0.05) using the R package Ballgown (R Foundation for Statistical Computing, Vienna, Austria). All DEGs were mapped to GO terms and KEGG pathways. Significantly enriched GO terms and KEGG pathways in DEGs were identified by hypergeometric tests with Bonferroni correction; *p* ≤ 0.05 was defined as the threshold.

The RNA samples used for RNA-seq were also used for qRT-PCR analysis. The first-strand cDNA was synthesized using a cDNA synthesis kit (Vazyme, Nanjing, China). Nine DEGs involved in chloroplast development and photosynthesis and three candidate genes in the region were selected for evaluation. Gene-specific primers were designed with Primer Premier Software v.5.0 (Premier Biosoft, Palo Alto, CA, USA) (Table S4), while the actin gene was used as an internal control. An amount of 2 μL cDNA (1:50 dilution), 25 μL of 2× Ultra SYBR Mix (CWBIO, Beijing, China), and 1 µL of each primer (100 nM final concentration) formed the 50-μL reaction, followed by the program of 95 °C for 10 min, and then 40 cycles of 95 °C for 15 s and 60 °C for 1 min. A melting curve analysis (55–95 °C) was performed at 95 °C for 15 s, 60 °C for 1 min, 95 °C for 15 s, and 60 °C for 15 s. Experiments were performed using a QuantStudio 6 PCR system (Thermo Fisher Scientific, Waltham, MA, USA) with three independent biological replicates. Relative expression level was calculated with the 2^−ΔΔCt^ method [42].

### 4.9. Statistical Analysis

Part of the statistical analyses in this study were performed using Student’s *t*-test. Values were considered as significantly different with *p* < 0.05 (*). For the rest, analysis of variance (followed by Duncan’s test) was used to test differences between samples, with *p* < 0.05 considered statistically significant.

## 5. Conclusions

The present study characterized a novel variegated leaf mutant ‘JC007-2B’ and revealed the molecular genetic mechanism of this unique variegated leaf phenotype in ornamental kale, which was featured by green margins and white centers. Chloroplast structure and photosynthetic pigments content in the white sectors of variegated leaves were abnormal. The *BoVl* locus responsible for the mutant phenotype was fine-mapped to a 6.74 Kb interval on chromosome C03. Based on the sequence variation and differential expression, *Bo3g002080*, which is homologous to *Arabidopsis MED4*, was the most likely candidate gene for *BoVl*. However, the function of *MED4* in affecting chloroplast development or determining pigment distribution has not been reported yet. To gain insights into the molecular mechanism underlying leaf variegation under the control of *BoVl*, we performed comparative transcriptome analysis between the green margins (S3_S) and the white centers (S3_C). Relevant pathways and DEGs involved in carotenoid biosynthesis, chloroplast, thylakoid membrane, and photosynthesis were significantly enriched. Our research provides a new gene resource for cultivar breeding in ornamental kale and contributes to uncovering the molecular genetic mechanism underlying variegated leaf formation.

## Figures and Tables

**Table 1:** Inheritance analysis of the variegated leaf trait in the populations constructed by the two parents in *Brassica oleracea*.

Population	Total	Variegated Leaves	Green Leaves	Segregation Ratio	χ^2^
P1 (‘JC007-2B’)	50	50	0		
P2 (‘BS’)	30	0	30		
P1 × P2	50	50	0		
P2 × P1	45	45	0		
(P1× P2) × P1	82	82	0		
(P1 × P2) × P2	24	11	13	0.846:1	0.17
F2	117	90	27	3.333:1	0.23

**Table 2:** The quality clean reads of two pools were yielded via BSR-seq.

Samples	Reads	Bases	Q20 (%)	Q30 (%)	GC (%)	N (ppm)
Var	49,679,016	7,367,237,292	97.80	94.16	47.64	11.27
CK	46,659,032	6,919,343,049	97.75	94.06	47.95	11.14

**Table 3:** The localization of chromosome regions related to the variegated leaf gene *BoVl.*

Chromosome	Start Position	Stop Position	Total SNV Numbers	Length	Total DEGs Numbers
C03	37,199	2,499,485	137	2,462,286	22
C03	3,047,605	4,030,163	12	982,558	5
C09	48,314,708	49,461,537	24	1,146,829	0

## Data Availability

The datasets of transcriptome analysis used in this research have been deposited in the Gene Expression Omnibus (GEO) under the accession number GSE112493. The datasets of BSR-seq can be found in the NCBI sequence reads archive (SRA) database under BioProject No. PRJNA900045.

## Supplementary Materials

The following supporting information can be downloaded at: https://www.mdpi.com/article/10.3390/ijms232314853/s1. Figure S1: Alignments of nucleotide sequences of *Bo3g002070* in ‘BS’ and ‘JC007-2B’; Figure S2: Alignments of nucleotide sequences of *Bo3g002090* in ‘BS’ and ‘JC007-2B’; Figure S3: Nucleotide and amino acid sequence alignments of *Bo3g002080* in ‘BS’ and ‘JC007-2B’; Figure S4: Nucleotide sequences alignments of *Bo3g002080* in two parents, F_2_ recombinant plants and DH line plants; Figure S5: Hydrophobicity and the protein structure of the candidate gene; Figure S6: The validation and expression analysis of another 9 randomly selected genes using qRT-PCR; Table S1: Data statistics and quality control of RNA-Seq; Table S2: Reference genome alignment reads statistics; Table S3: Primer sequences for *Bo3g002070*, *Bo3g002080*, *Bo3g002090*. Table S4: Primers used to validate expression patterns determined by transcriptome profiling, and primers for the qRT-PCR of candidate genes.
