# Supplementary material for: Fine Mapping of BoVl Conferring the Variegated Leaf in Ornamental Kale (Brassica oleracea var. acephala)
Source: Int J Mol Sci. 2022 Nov 27;23(23):14853. doi: 10.3390/ijms232314853 (PMC9739133; doi:10.3390/ijms232314853)
Supplement: Supplementary file 1 [file ijms-23-14853-s001.zip › ijms-1998883-supplementary.pdf]

Fine mapping of *BoVl* conferring variegated leaf in ornamental kale (*Brassica oleracea* var. *acephala*)

Supplementary information: Figure S1-S6 and Table S1-S4.

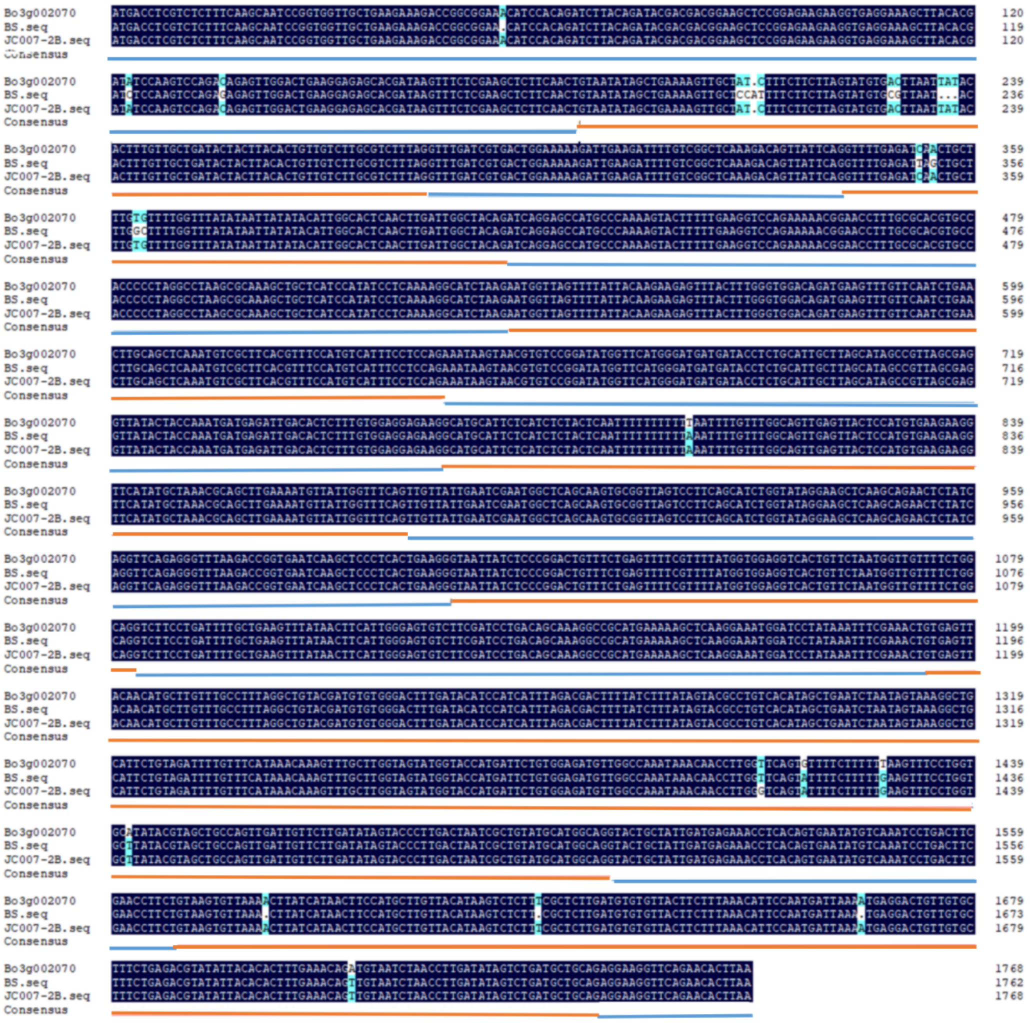

Figure S1. Alignments of nucleotide sequences of *Bo3g002070* in 'BS' and 'JC007-2B'. Note: *Bo3g002070* is the reference genome sequence of cabbage; Blue underline indicates exon; Red underline indicates intron.

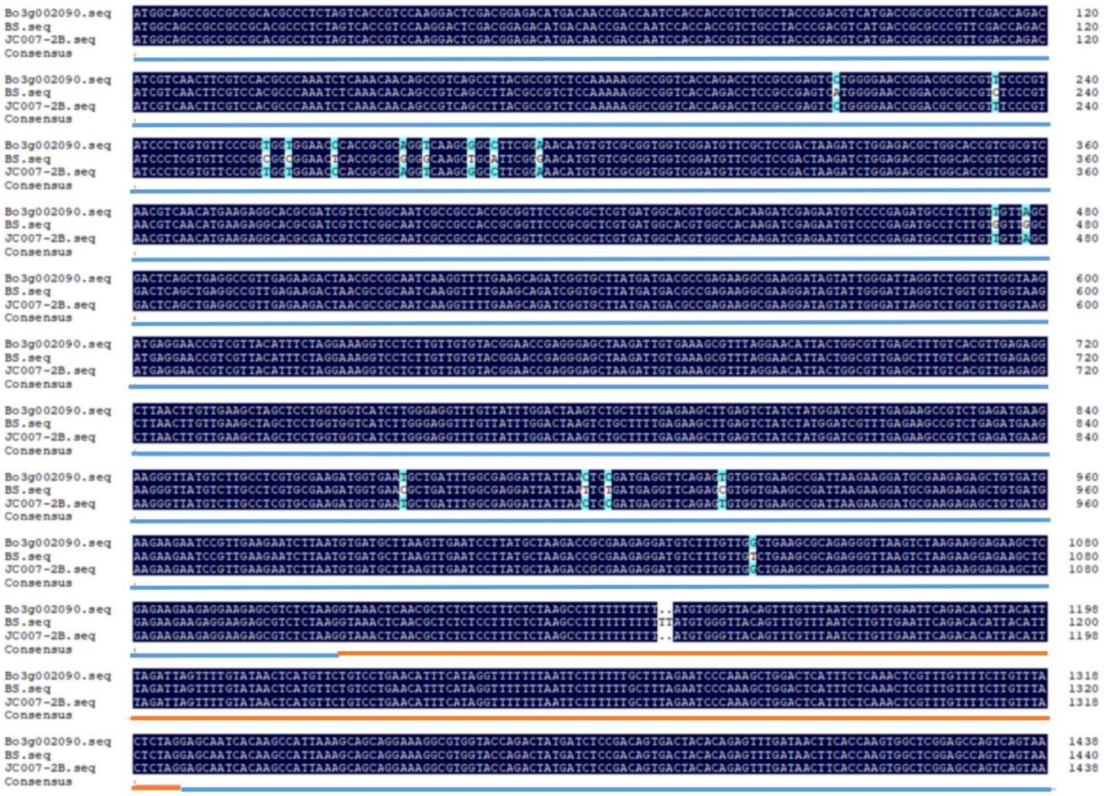

Figure S2. Alignments of nucleotide sequences of *Bo3g002090* in 'BS' and 'JC007-2B'. Note: *Bo3g002090* is the reference genome sequence of cabbage; Blue underline indicates exon; Red underline indicates intron.

a

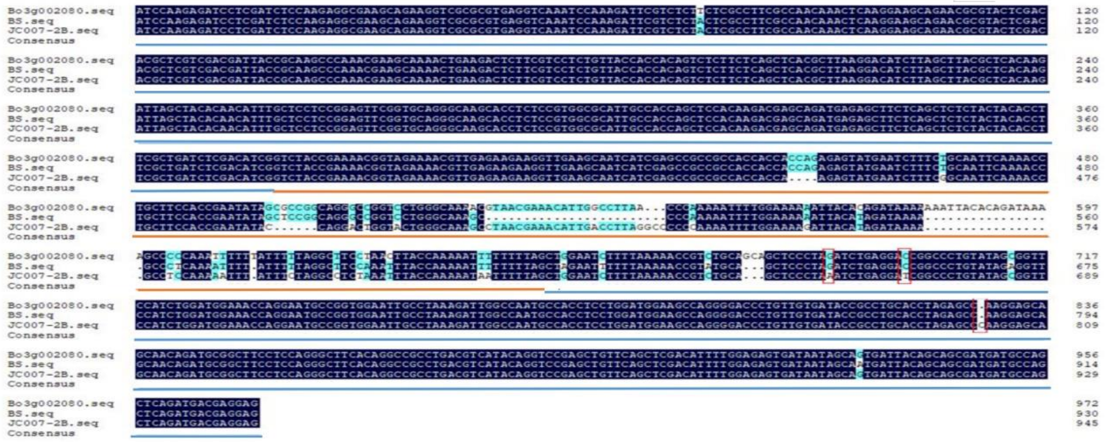

b

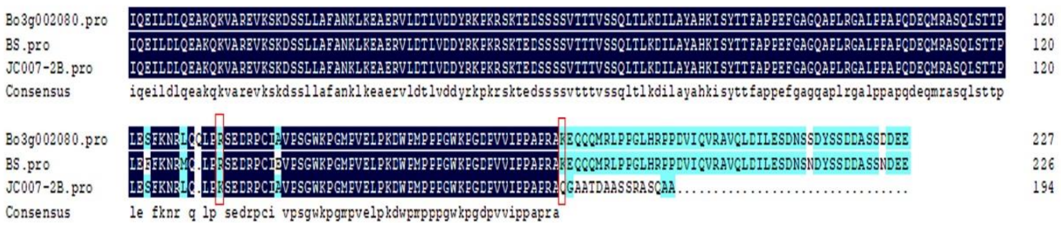

Figure S3. Nucleotide and amino acid sequence alignments of *Bo3g002080* in 'BS' and 'JC007-2B'. (a) Nucleotide sequences alignments of *Bo3g002080* in 'BS' and 'JC007-2B'. Blue underline represents exon; Red underline represents intron. (b) Amino acid sequence alignment of *Bo3g002080* in 'JC007-2B' and 'BS'. 'Bo3g002080' is the reference genome sequence of from non-variegated phenotype cabbage. Red boxes represent base substitutions.

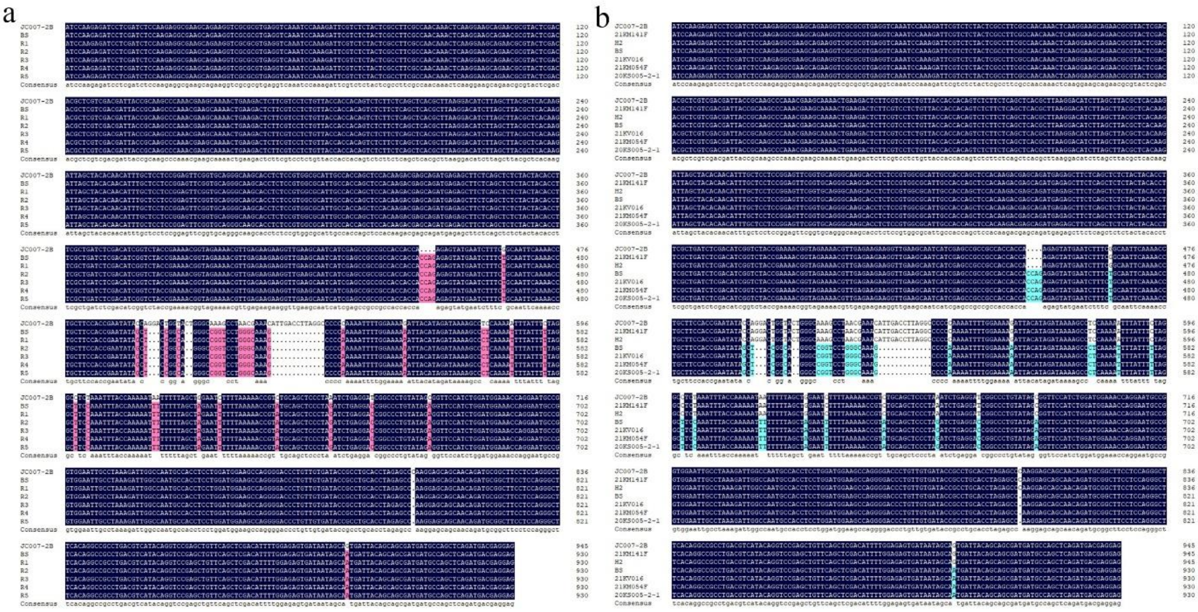

Figure S4. Nucleotide sequences alignments of *Bo3g002080* in two parents, F_2 recombinant plants and DH line plants. (a) Nucleotide sequences alignments of *Bo3g002080* among two parents and recombinant plants. R1-R5 represent the five recombinant plants that identified by Q3I-8 and Q3I-15; (b) Nucleotide sequences alignments of *Bo3g002080* among two parents and DH lines. 'JC007-2B', 'H2' and '21KM141F' were with variegated leaf, while the leaf phenotype of 'BS', '21KV016', '21KM054F' and '20KS005-2-1' was normal.

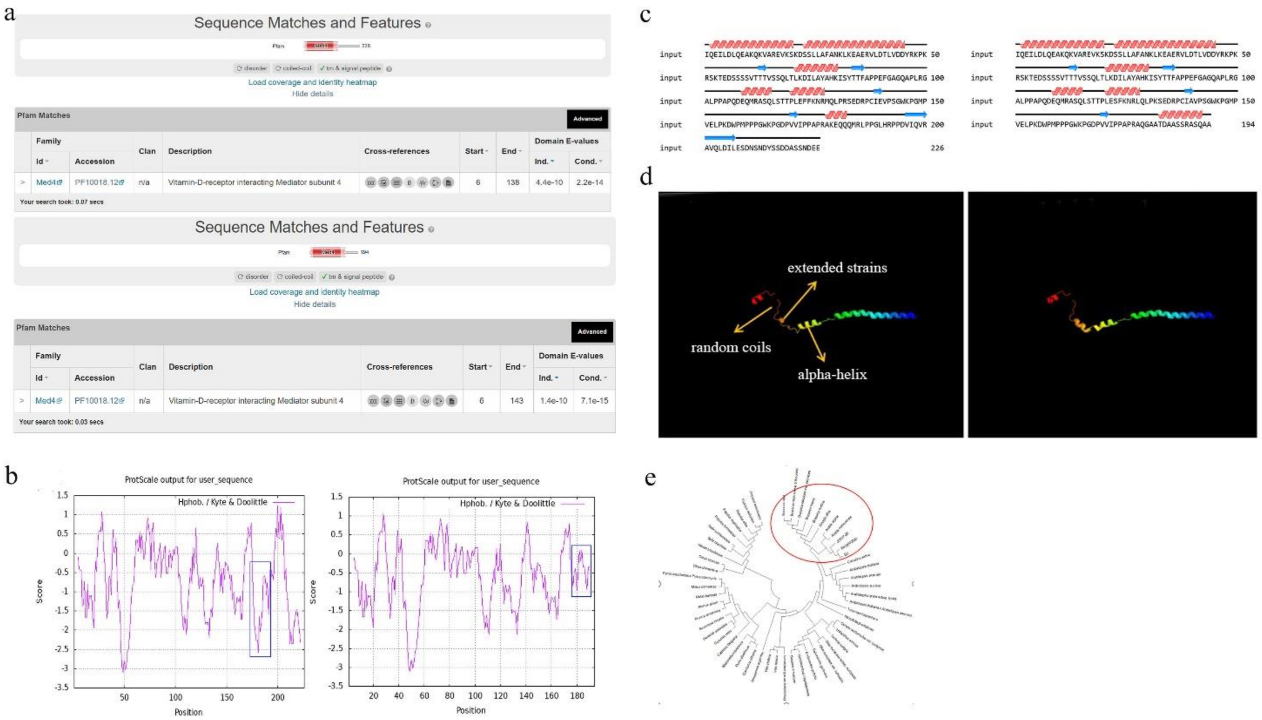

Figure S5. Hydrophobicity and the protein structure of the candidate gene. (a) Conserved domain analysis, the upper figure was the conserved domain analysis of 'BS', and the down figure was the conserved domain analysis of 'JC007-2B'; (b) Hydrophobicity analysis of 'BS' and 'JC007-2B' protein. The left figure was the hydrophobicity analysis of 'BS', and the right figure was the hydrophobicity analysis of 'JC007-2B'; (c) The predicted secondary structure of 'BS' and 'JC007-2B' protein. The left figure was the predicted secondary structure of 'BS', and the right figure was the predicted secondary structure of 'JC007-2B'. The red asterisk represents the position of 132 amino acids, and the green asterisk represents the position of 179 amino acids; (d) The predicted secondary structure of 'BS' and 'JC007-2B' protein. The left figure was the predicted secondary structure of 'BS', and the right figure was the predicted secondary structure of 'JC007-2B'; (e) Phylogenetic tree of *B03g002080*, 'BS' and 'JC007-2B' in ornamental kale and other species. *Brassica rapa* (RID43011.1); *Brassica cretica* (KAF2535840.1); *Brassica oleracea* var. *oleracea* (XP013611601.1); *Brassica napus* (KAH0908033.1); *Arabis alpina* (KFK24735.1); *Brassica rapa* subsp. *trilocularis* (VVB12997.1); *Camelina sativa* (XP010490754.1); *Tarenaya hassleriana* (XP010553019.1); *Prunus armeniaca* (CAB4304152.1); *Capsella rubell*a (XP006287800.1); *Carpinus fangiana* (KAE8099520.1); *Arabidopsis arenosa* (CAE6085689.1); *Arabidopsis suecica* (KAG7553896.1); *Arabidopsis thaliana* (AAP40461.1); *Arabidopsis lyrata* subsp. *Lyrata* (XP020879558.1); *Camelina sativa* (XP010423809.1); *Arabidopsis thaliana* x *Arabidopsis arenosa* (KAG7549282.1); *Helianthus annuus* (XP022039233.1); *Sesamum indicum* (XP011077385.1); *Citrus clementina* (XP006428900.1); *Rehmannia glutinosa* (ALO61893.1); *Salix brachista* (KAB5519807.1); *Salix dunnii* (KAF9664990.1); *Olea europaea* subsp.*Europaea* (CAA3013307.1); *Durio zibethinus* (XP022748477.1); *Olea europaea* var. *Sylvestris* (XP022846688.1); *Sinapis alba* (KAF8088602.1); *Populus trichocarpa* (XP_002322847.1); *Populus deltoides* (KAH8485176.1); *Salix suchowensis* (KAG5253153.1); *Prunus armeniaca* (CAB4270352.1); *Populus euphratica* (XP011048421.1); *Handroanthus impetiginosus* (PIN03719.1); *Nicotiana attenuata* (XP019238085.1); *Lactuca sativa* (XP023762142.1); *Citrus sinensis* (XP006480577.1); *Malus baccata* (TQE13352.1); *Populus tomentosa* (KAG6744068.1); *Populus alba* (XP034910512.1); *Pyrus ussuriensis* x *Pyrus communis* (KAB2597909.1); *Malus domestica* (RXI07622.1); *Cucumis sativus* (XP004142109.1); *Corchorus olitorius* (OMO73465.1); *Cynara cardunculus* var. *Scolymus* (XP024990234.1); *Hevea brasiliensis* (XP021666870.1); *Cucumis melo* (XP008449288.1); *Rhododendron williamsianum* (KAE9447899.1); *Prunus avium* (XP021822650.1); *Vitis vinifera* (XP002273310.1); *Benincasa hispida* (XP038882754.1); *Vitis riparia* (XP034693987.1); *Vitis vinifera* (RVW98737.1); *Erythranthe guttata* (XP_012842735.1).

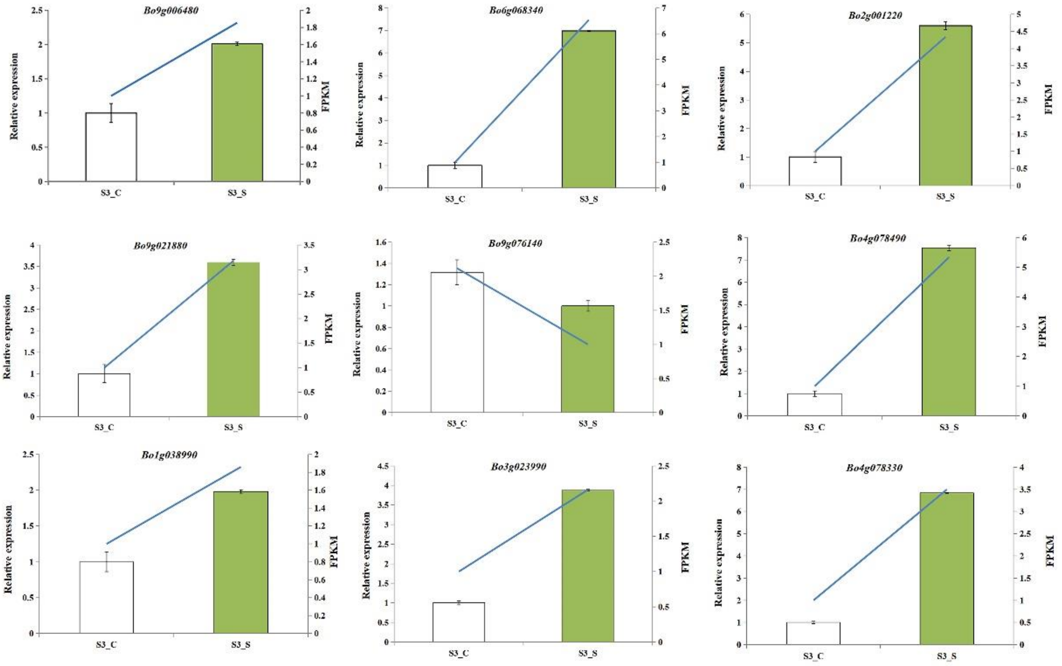

Figure S6. The validation and expression analysis of another 9 randomly selected genes using qRT-PCR.

Table S1 Data statistics and quality control of RNA-Seq.

Sample	Raw Data	Valid Data	Valid Ratio（reads）	Q20%	Q30%	GC content%
S3_S_1	378，777，42	373，854，02	98.70	99.67	96.23	47
S3_S_2	478，043，84	472，213，44	98.78	99.64	96.11	47
S3_S_3	504，932，20	499，029，14	98.83	99.68	96.25	46.5
S3_C_1	521，933，28	516，489，84	98.96	99.77	96.46	46.5
S3_C_2	574，185，18	567，177，70	98.78	99.64	95.92	46.5
S3_C_3	487，531，74	483，011，34	99.07	99.76	96.12	47

Table S2 Reference genome alignment reads statistics.

Sample	Valid reads	Mapped reads	Unique Mapped reads	Multi Mapped reads	PE Mapped reads Pair-end
S3_S_1	373,854,02	334,514,59（89.48%）	245,067,02（65.55%）	894,475,7（23.93%）	297,076,06（79.46%）

S3_S_2	472,213,44	419,359,98（88.81%）	309,063,02（65.45%）	110,296,96（23.36%）	372,903,16（78.97%）
S3_S_3	499,029,14	445,226,67（89.22%）	328,172,86（65.76%）	117,053,81（23.46%）	402,526,62（80.66%）
S3_C_1	516,489,84	459,860,60（89.04%）	336,564,87（65.16%）	123,295,73（23.87%）	418,153,94（80.96%）
S3_C_2	567,177,70	501,622,41（88.44%）	367,100,13（64.72%）	134,522,28（23.72%）	444,724,58（78.41%）
S3_C_3	483,011,34	429,720,63（88.97%）	310,603,44（64.31%）	119,117,19（24.66%）	391,313,86（81.02%）

Table S3 Primer sequences for *Bo3g002070*, *Bo3g002080*, *Bo3g002090.*

Gene	Primers	Sequences（5’ to 3’）
Bo3g002070	2070-1-L	ATGACCTCGTCTCTTTCAAGCAAT
	2070-1-R	CTTCTCCTCCACAAAGAGTGTCAAT
	2070-2-L	CGGATATGGTTCATGGGATGA
	2070-2-R	TTCGAAATTTATAGGATCCATTTCC
	2070-3-L	CGGATATGGTTCATGGGATGA
	2070-3-R	ATAAGTGTTCTGAACCTTCCTCTGC
Bo3g002080	2080-1-L	GGGATATTAAATCGTATTGGTCTGG
	2080-1-R	TGACTTACTCCTCGTCATCTGAGC
	2080-2-L	TAAACGCACAGCCACACTCCT
	2080-2-R	TCCAAATAACAAACCTCCCAAGAT
Bo3g002090	2090-1-L	AAGCGTTTAGGAACATTACTGGCG
	2090-1-R	ATAACCAAAATCTTATATGAGACGACAA

Table S4 Primers used to validate expression patterns determined by transcriptome profiling, and primers for the qRT-PCR of candidate genes.

Markers	Forward primer sequences (5′→3′)	Reverse primer sequence (5′→3′)	Gene
CAD	GCCGTACTTGTCTCTGCTTAG	TCCTCTGTTTCCTTCATGCTC	*Bo7g116770*
PSAO	GCTCTCACTTCTCAGTTCTGG	ATCCGATTTGTCCGAAGGTG	*Bo8g112500*
LHCA4	CTTAACTTCGCTCCTACGCTC	TCACGAACCCTAAGAATGCC	*Bo3g134510*
PSBQ	GGATAGATGGTCCTCTTCCAAC	CTTCCTTTGGTCCCGATGTC	*Bo1g158910*
LHCB4	GAAAGGTGGAGCTAGTGGATG	GGGTACAAACGCTTCTCAGAG	*Bo2g001220*
LHCB6	AGAATTTCGCTGTATCCCGG	ACTTTCAACCTCTCCAGCTTC	*Bo8g066580*
PSAE	AATCCTACTGGTTCAAGAGCG	TCTTCAATCTCGTCCAGTGC	*Bo8g054500*
PSAN	ATGTTGATGGGTCTAGTGGAAG	GAGGTATTCATCGAAGACGCTA G	*Bo3g102750*
GLK1	AGGAAAAGGGTCTTGAGGTTC	GGTGAGTGCTTACAGGTACATG	*Bo7g003410*
YBo3g002070	TCGTCTCTTTCAAGCAATCCG	CTGTCTGGACTTGGATATCGTG	*Bo3g002070*
YBo3g002080	CAAACTCAAGGAAGCAGAACG	GTGGTAACAGAGGACGAAGAG	*Bo3g002080*
YBo3g002090	TGGTCATCTTGGGAGGTTTG	GCCAAATCAGCATTCACCATC	*Bo3g002090*
Actin	GGTCGTGACCTTACTGATTACCT CA	GAAGTCTCCATCTCCTGCTCGT	*Bo1g116200*
